# Supplementary material for: Genome-Wide Characterization of the INDETERMINATE DOMAIN (IDD) Zinc Finger Gene Family in Solanum lycopersicum and the Functional Analysis of SlIDD15 in Shoot Gravitropism
Source: Int J Mol Sci. 2024 Sep 27;25(19):10422. doi: 10.3390/ijms251910422 (PMC11476865; doi:10.3390/ijms251910422)
Supplement: Supplementary file 1 [file ijms-25-10422-s001.zip › ijms-3200579-supplementary.pdf]

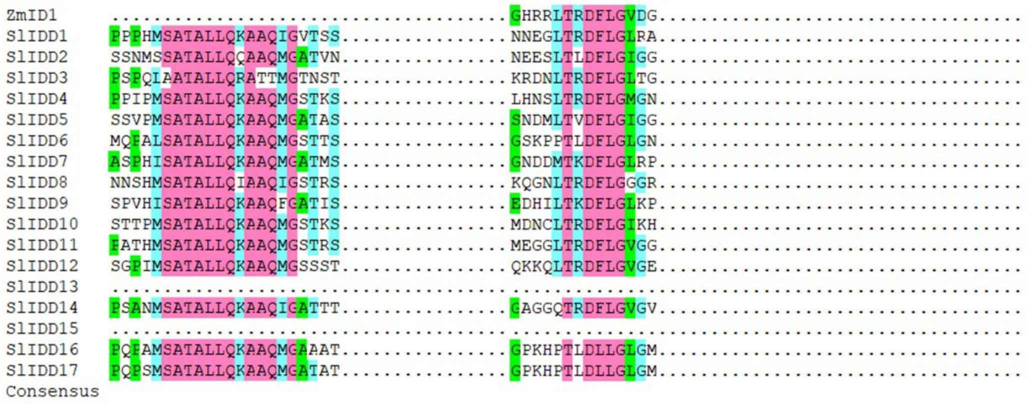

Figure S1. Multiple sequence alignment of two C-terminal structures of SlIDD protein and ZmID1.The C-terminal conserved TR/L/QDFLG domain and MSATALLQKAA domain of 17 SlIDD and ZmID1 protein sequences was aligned by DNAMAN.

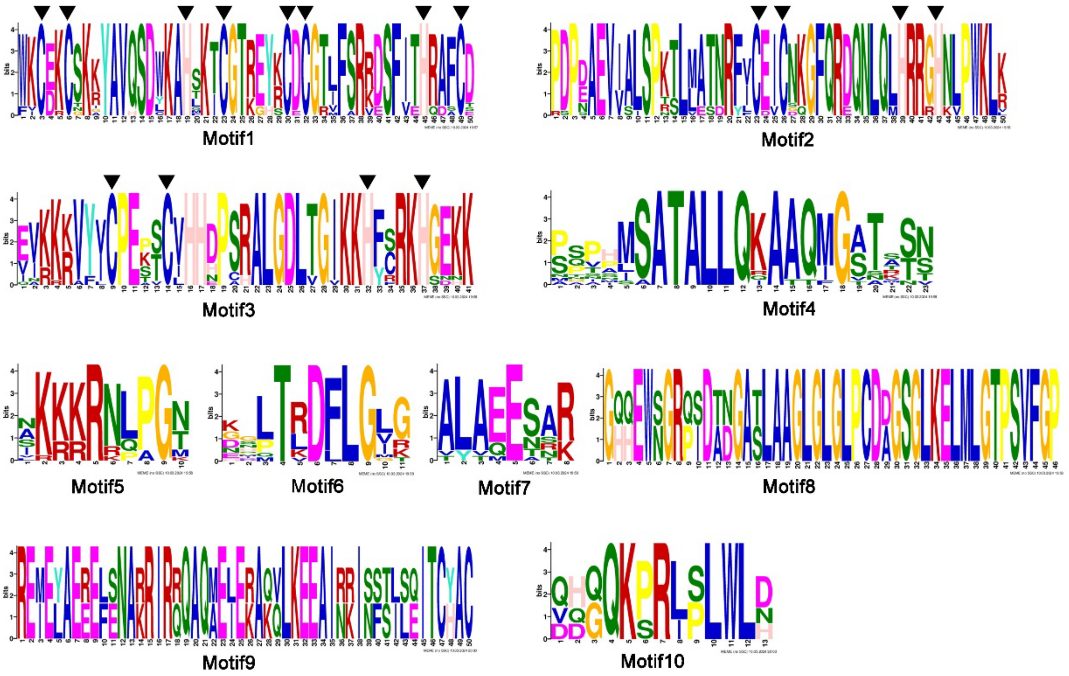

Figure S2. Details of ten selected motifs. This figure utilizes the MEME tool to conduct motif analysis on the SlIDD proteins and generate the corresponding sequence logo. The height of each base at each position in the logo represents its frequency of occurrence within the motif. The black solid inverted triangle marks the amino acids in the conserved ID-domain.

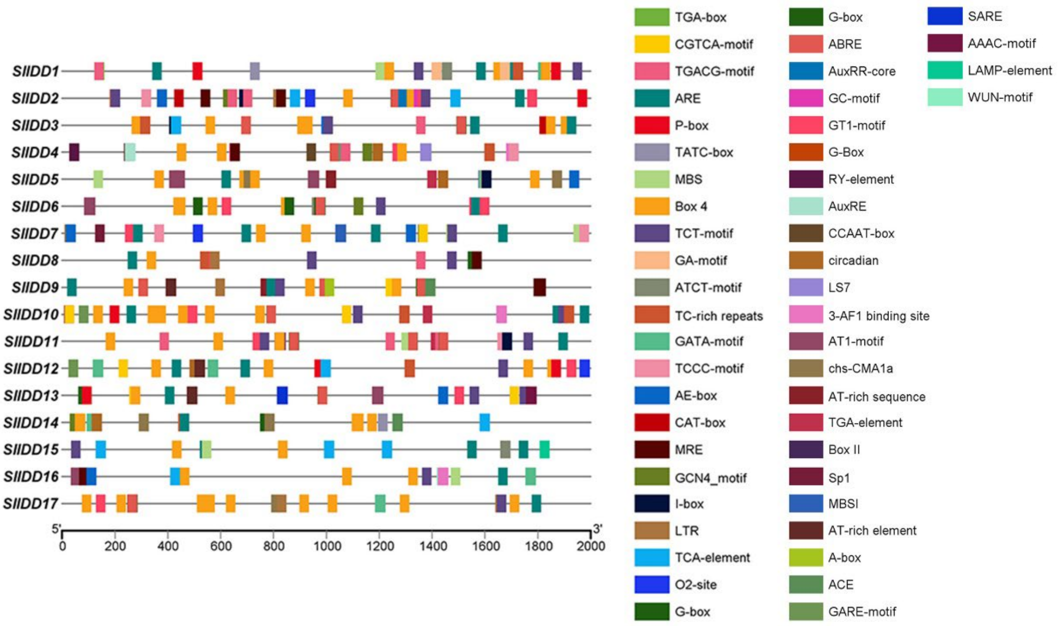

Figure S3. Distribution of *cis*-acting elements of *SlIDD* gene family in tomato. Vertical bars display the positional distribution of the projected *cis*-acting elements on the *SlIDD* promoters. PlantCARE was used to analyze the promoter sequences (2000 bp) of 17 *SlIDD* genes. In this legend, each color represented different *cis*-elements.

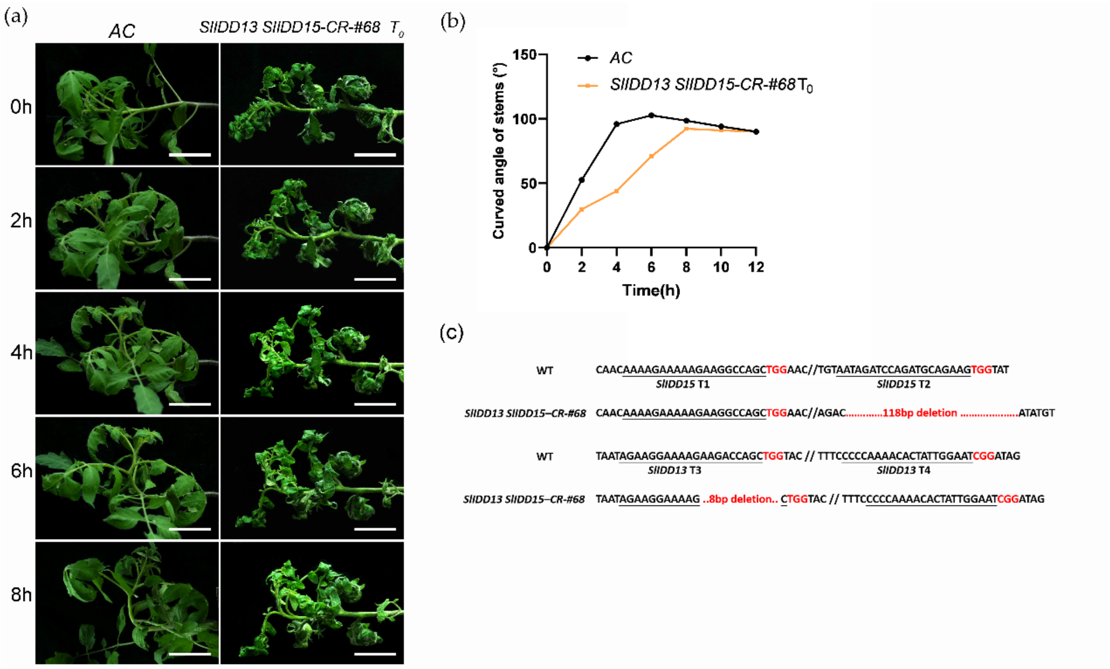

Figure S4. *SlIDD15* and *SlIDD13* knockout mutant plant phenotype and mutation site. (a)Phenotypes of wild-type *AC* and *SlIDD13 SlIDD15-CR-#68* mutant under gravity stimulation in T0 generation. (b) Statistical analysis of the stem inclination angle after gravity direction change in individual *SlIDD13 SlIDD15-CR-#68* mutant. Scale bar = 5 cm, arrow g indicates the direction of gravity. (c)Schematic diagram of the mutation site in *SlIDD13 SlIDD15-CR-#68* mutant.
